# Supplementary material for: Structure of a AAA+ unfoldase in the process of unfolding substrate
Source: eLife. 2017 Apr 8;6:e25754. doi: 10.7554/eLife.25754 (PMC5423775; doi:10.7554/eLife.25754)
Supplement: Supplementary file 1. — Cryo-EM data acquisition, processing, and atomic model statistics. DOI: http://dx.doi.org/10.7554/eLife.25754.015 [file elife-25754-supp1.pdf]

**Supplementary file 1.** Cryo-EM data acquisition, processing, and atomic model statistics.

| **Data Collection** | **ΔN-VAT** |
|---|---|
| Electron Microscope | Tecnai F20 |
| Camera | K2 Summit |
| Voltage | 200 kV |
| Nominal Magnification | 25,000× |
| Calibrated physical pixel size | 1.45 Å |
| Total exposure | 35 electrons/Å$^2$ |
| Exposure rate | 5 electrons/pixel/s |
| Number of frames | 30 |
| Defocus range | 1.7-2.9 μm |
| **Image Processing** | |
| Motion correction software | *alignparts_lmbfgs* |
| CTF estimation software | CTFFIND4 |
| Particle selection software | Relion 1.4 |
| Micrographs used | 246 |
| Particles selected | 171,381 |
| 3D map classification and refinement software | cryoSPARC |
| Particles contributing to final map (stacked-rings) | 75,205 |
| Applied symmetry (stacked-rings) | C6 |
| Applied B-factor (stacked-rings) | 199.5 Å$^2$ |
| Global resolution (FSC = 0.143) (stacked-rings) | 3.9 Å |
| Particles contributing to final map (substrate-bound) | 13,238 |
| Applied symmetry (substrate-bound) | C1 |
| Applied B-factor (substrate-bound) | 218.8 Å$^2$ |
| Global resolution (FSC = 0.143) (substrate-bound) | 4.8 Å |
| **Model Building** | |
| Modeling software | *Coot, Phenix* |
| Number of residues built | 3264 |
| RMS (bonds) | 0.0037 |
| RMS (angles) | 0.96 |
| Ramachandaran outliers | 0.00 % |
| Ramachandran favoured | 96.49 % |
| Clashscore | 20.17 |
| MolProbity score | 2.59 |
| EMRinger score | 1.17 |
